# Consumers’ Drivers of Perception and Preference of Fermented Food Products and Beverages: A Systematic Review

**DOI:** 10.3390/foods14050713

**Published:** 2025-02-20

**Authors:** Sergio Erick García-Barón, Rosa Pilar Carmona-Escutia, Enrique J. Herrera-López, Doris Arianna Leyva-Trinidad, Anne Gschaedler-Mathis

**Affiliations:** 1 ESDAI, Universidad Panamericana, Augusto Rodin 498, Ciudad de México 03920, Mexico; sergiogarcia@up.edu.mx; 2 Unidad de Biotecnología Industrial, Centro de Investigación y Asistencia en Tecnología y Diseño del Estado de Jalisco, A.C., Camino Arenero 1227, El Bajío del Arenal, Zapopan 45019, Mexico; 3 Unidad de Tecnología Alimentaria, Centro de Investigación y Asistencia en Tecnología y Diseño del Estado de Jalisco, A.C., Av. Normalistas 800, Guadalajara 44270, Mexico; rcarmona@ciatej.mx; 4 Laboratorio para la Innovación en Bioelectrónica e Inteligencia Artificial, LINBIA, Unidad de Biotecnología Industrial, Centro de Investigación y Asistencia en Tecnología y Diseño del Estado de Jalisco, A.C., Camino Arenero 1227, El Bajío del Arenal, Zapopan 45019, Mexico; 5 Coordinación de Desarrollo Regional, Centro de Investigación en Alimentación y Desarrollo, A.C. Carretera Gustavo Enrique Astiazarán Rosas 46, Hermosillo 83304, Mexico; doris.leyva@ciad.mx

**Keywords:** consumer perception, consumer behavior, food choice, dairy products, plant-based, meat products

## Abstract

The fermentation of food products is a transformation and preservation process in which different metabolites are generated, contributing to consumer health. In this sense, this systematic review aims to analyze the factors that guide the perception and preference for fermented foods. In addition, different perspectives are proposed based on the findings. The systematic search was carried out in four databases: Emerald Insight, Science Direct, Wiley Online Library, and Multidisciplinary Digital Publishing Institute. The keywords used were (Title/Abstract): fermented products, overall liking, purchase intention, expectations, emotions, interculturality, perception, and consumers. Ninety-two articles (n = 92) were selected and classified. The factors identified were (1) biological and physiological, (2) extrinsic product characteristics, (3) intrinsic product characteristics, (4) psychological, (5) situational, and (6) sociocultural. Intrinsic product characteristics were the most relevant, while the situational factors were the least studied. Our main contribution was a multidisciplinary approach to addressing the different factors in an integrated way, allowing a broader perspective of both products and consumers. This approach could help the reader understand consumer behavior and propose product improvements.

## 1. Introduction

The fermentation process of food and beverages is one of the oldest transformation and conservation techniques developed by humanity [1,2]. The first evidence of the preparation and consumption of fermented food products and beverages (FFPBs) is vestiges of vessels found in archaeological sites in Asia around 8000 BC [3]. Over time, FFPBs have gained social, cultural, economic, and gastronomic importance. Fermented products began to represent one-third of the population’s diet in the regions where they originated [4]. FFPBs include dairy products [5,6], meat products [7,8], beverages [9,10], and plant-based products [11,12,13]. During the fermentation process, the bioavailability of nutrients can be enhanced, stimulating probiotic and prebiotic properties, and improving nutritional properties and health benefits [3]. 

In this sense, FFPBs have been associated with different health benefits for the consumer, such as anti-diabetic properties [14], anti-hypertensive effects [15], inhibition of fat accumulation in adipose tissue [16], and modulation of the immune system [17].

Recently, it has been observed that consumers are increasingly aware of their diet and recognize that they can reduce the risk of diseases through a healthy lifestyle, including the diet; thus, the interest of consumers in healthy and natural foods, such as FFPBs, has increased [18,19]. Additionally, this type of product represents a sustainable alternative that contributes to reducing food waste and has the potential to generate value-added foods [1]. Likewise, in some cases, FFPBs reflect culinary traditions, as well as a strategy to encourage gastronomic tourism [2,3,9]. Mandha et al. [10] raised the need to study and understand consumer perceptions and preferences, where it is desired to identify the factors that influence consumer behavior and detect product parameters that require further development.

**Figure 1:**
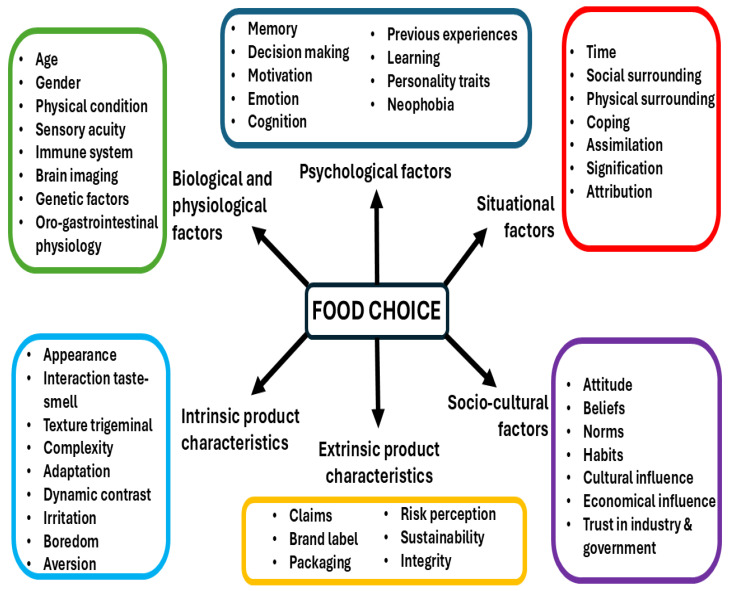
Factors and sub-factors influencing consumer behavior and food choice (adapted from Mojet [21]).

Food selection is a complex process involving interacting factors of different order and nature. Due to the complexity of the process of food consumption and selection, various models have been proposed to study and understand the procedure [20]. The transdisciplinary approach is the most suitable to build a holistic conceptual model that is used to explain food choice, and should be supported by empirical evidence from different areas of knowledge [20,21]. In line with this, Mojet [21] proposed a holistic model to identify and categorize the essential factors that influence eating and drinking behavior and food choice. The holistic model considers six factors: biological and physiological, extrinsic product characteristics, intrinsic product characteristics, psychological factors, situational factors, and sociocultural factors (Figure 1). The model considers the dynamic and complex interactions between food, consumers, and the environment, thus increasing its ecological validity.

This systematic review has two objectives: (i) to identify the factors that drive consumer perception and preference for fermented food and beverages products, and (ii) to classify the factors that influence the perception and consumption of fermented foods. Furthermore, the perspectives that can be gained from studying the drivers of perception and preferences within FFPBs are raised. Therefore, this review addresses the following research questions.

RQ1: What factors influence the perception and consumption of fermented food and beverages?

RQ2: What future implications do the factors that influence consumer behavior of fermented products and beverages have?

## 2. Materials and Methods

### 2.1. Search Process, Information Sources, and Search Strategy

To answer the research questions, a systematic review of the state of the art was adopted. A systematic review follows a pre-established inclusion to answer a research question or hypothesis [22]. The research was conducted in four databases: Emerald Insight, Science Direct, Wiley Online Library, and Multidisciplinary Digital Publishing Institute. The research followed the guidelines established by the Preferred Reporting Items for Systematic Review and Meta-Analysis (PRISMA) [23] using the following keywords (Title/Abstract): fermented foods, liking, purchase intention, expectations, emotions, cross-cultural, perception, and consumers. It identified 3234 documents. In our case, it was not necessary to register the search protocol.

### 2.2. Inclusion Criteria

Only original articles written in English and published between January 2014 and June 2024 were considered. According to the search parameters, 2353 articles were obtained.

### 2.3. Exclusion Criteria

**Figure 2:**
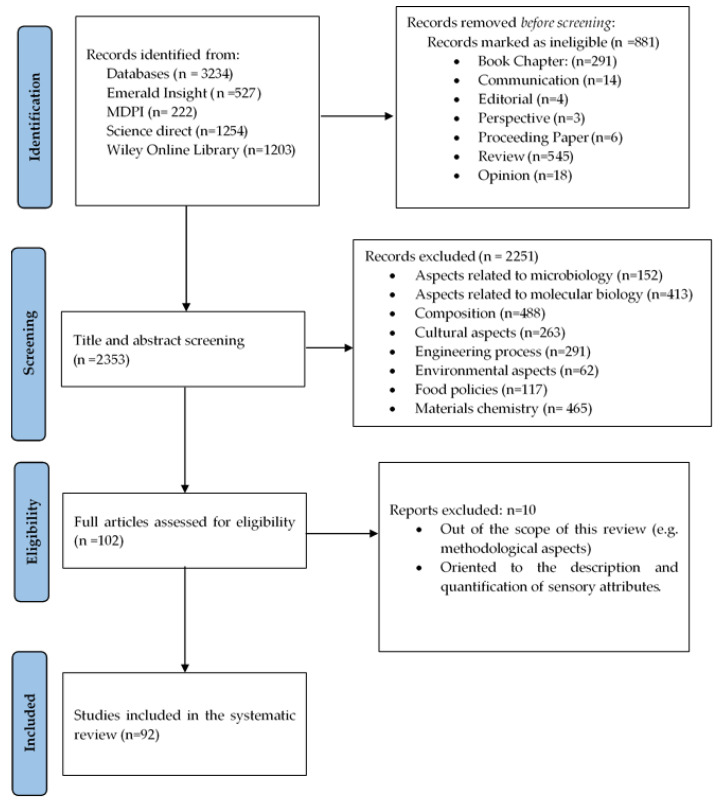
PRISMA flowchart of the article search and selection process.

As a result of the screening, 2251 papers were excluded. A total of 488 were oriented to the composition of fermented and non-fermented products, 263 studied cultural aspects of food, 291 papers focused on process engineering, 152 were associated with microbiological issues, 413 dealt with the molecular biology of the processes, 465 studied the chemistry of materials, 117 studies dealt with food policy, and 62 were related to environmental issues in food products. Subsequently, 102 articles were analyzed for the eligibility stage, of which, 10 were excluded (6 focused on the description and quantification of sensory attributes, 4 oriented to methodological aspects). Finally, 92 papers met the inclusion criteria and were considered in the systematic review. The articles were organized by reference, factor, and sub-factors (details of the selected articles are shown in the Supplementary Materials in Tables S1–S6). It is worth mentioning that a single article may address several factors and sub-factors. In this regard, Figure 2 shows a flow chart with the different stages carried out during the systematic review, including the number of articles found, the articles included and excluded, and the exclusion criteria.

### 2.4. Article Selection

Before carrying out the selection process, the researchers agreed on the definitions of the factors to facilitate classification. The process of selecting the articles was carried out independently by the two main authors of the study. The titles of the articles were then screened for admissibility. The full texts of the selected studies were then analyzed to determine their eligibility. An article was selected if it addressed at least one of the previously defined factors. When discrepancies or disagreements arose during the selection process regarding the type of factor, they were addressed through discussion and consensus among the reviewers, requiring a third reviewer.

### 2.5. Data Collection

Once the data were extracted independently, the information was reviewed by the other team members. The selected articles were organized and classified using the Mojet model [21] as a reference. Finally, the Cochrane Risk of Bias-2 tool [24] was used to assess the quality of the studies considering the risk of bias.

### 2.6. Data Analysis

First, the relevance of the factors and sub-factors studied was determined based on the number of articles that listed them. The Chi-squared test of the K proportions test (Marascuilo procedure) was performed to determine significant differences (*p* < 0.05) between factors. In the case of the sub-factors, comparisons were made within each factor. The analysis was performed using XLSTAT 2024 software (Addinsoft, Paris, France).

A network map showing the associative interaction between the sub-factors was designed using the software VOSviewer 1.6.20 (Leiden, The Netherlands). First, a tab-separated TXT file contained information about the sub-factors such as ID, label, description, weight, *x*, *y*, cluster, and RGB values. The label lists the sub-factors in alphabetical order, associated with the factor given in the description column, while the ID is the consecutive number of sub-factors. The weight is the attribute describing the frequency of a specific sub-factor; the higher the weight of an item, the more prominent the size of the sub-factor. The variables *x* and *y* represent the location coordinates in the main window of the software VOSviewer. Clusters are one of the six factors, i.e., biological and physiological, extrinsic product characteristics, etc. A second TXT file included the list of coordinates for the (*x*, *y*) pair that interconnects two sub-factors, considering the frequency of each connection. RGB describes the red, green, and blue values of each cluster.

## 3. Results and Discussion

According to the inclusion criteria, only 3.2% of the articles were considered after a review of the title and abstract (102 articles/3234 articles). This result suggests that issues related to consumption would be less addressed compared to the other topics related to fermented foods. Finally, once the relevance of the papers was confirmed, 90.2% of the articles were considered (92 articles/102 articles). The Cochrane Risk of Bias-2 tool showed that the risk determined in the reviewed articles was low. To do so, the five domains included in the tool were considered (Domain 1: Risk of bias arising from the randomization process; Domain 2: Risk of bias due to deviations from the intended interventions; Domain 3: Missing outcome data; Domain 4: Risk of bias in measurement of the outcome; Domain 5: Risk of bias in selection of the reported result; and Overall risk of bias).

### 3.1. Factors Driving Perception, Preference, and Consumption of Fermented Products and Beverages

**Figure 3:**
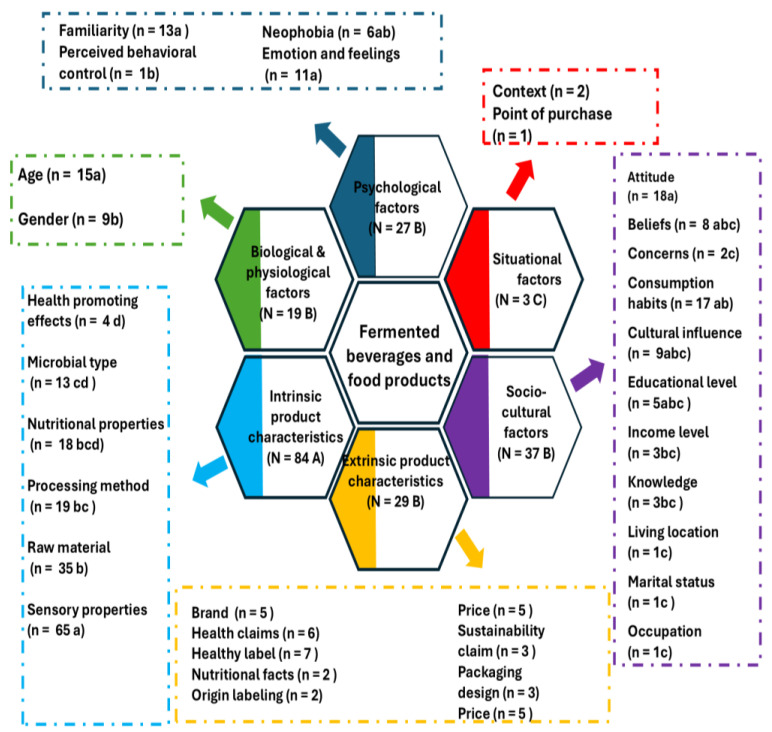
Factors and sub-factors that determine consumer perception and preference for fermented products and beverages. N is the number of articles that addressed a given factor, and n addressed a given sub-factor. Capital letters indicate significant differences between factors (*p* < 0.05). Lowercase letters indicate significant differences between sub-factors within each factor (*p* < 0.05). Differences were calculated using the Chi-squared test (*p* < 0.05) of the K proportions test using the Marascuilo procedure.

The factors that guide the preference and perception of FFPBs identified in the systematic search were classified into six factors, following Mojet’s model [21] as a reference (Figure 3): biological and physiological, extrinsic product characteristics, intrinsic product characteristics, psychological, situational, and sociocultural factors. According to K proportions analysis, differences were found in the number of articles studying the factors (*p* < 0.05) (details of the comparisons between the factors and their corresponding sub-factors are reported in the Supplementary Materials Tables S7–S13). Intrinsic product characteristics were the most addressed factor, followed by sociocultural factors, extrinsic product characteristics, psychological factors, and biological and physiological factors.

The results suggest that intrinsic characteristics were the most important factors influencing consumer behavior of FFPBs. In contrast, situational factors were the least studied. Therefore, it is necessary to investigate the role of this factor in the consumer behavior of FFPBs. Regarding the sub-factors, except for extrinsic characteristics and situational factors, significant differences were observed at the sub-factor level in the number of items studied (*p* < 0.05). It should be noted that in intrinsic product characteristics, the sensory characteristics of fermented products have been widely explored. In the case of sociocultural factors, attitude is the most studied sub-factor. Within the biological and physiological factors, the age sub-factor has been the most examined. Psychological factors, familiarity, and emotions and feelings were the most studied sub-factors.

### 3.2. Sub-Factor Network Analysis Map

**Figure 4:**
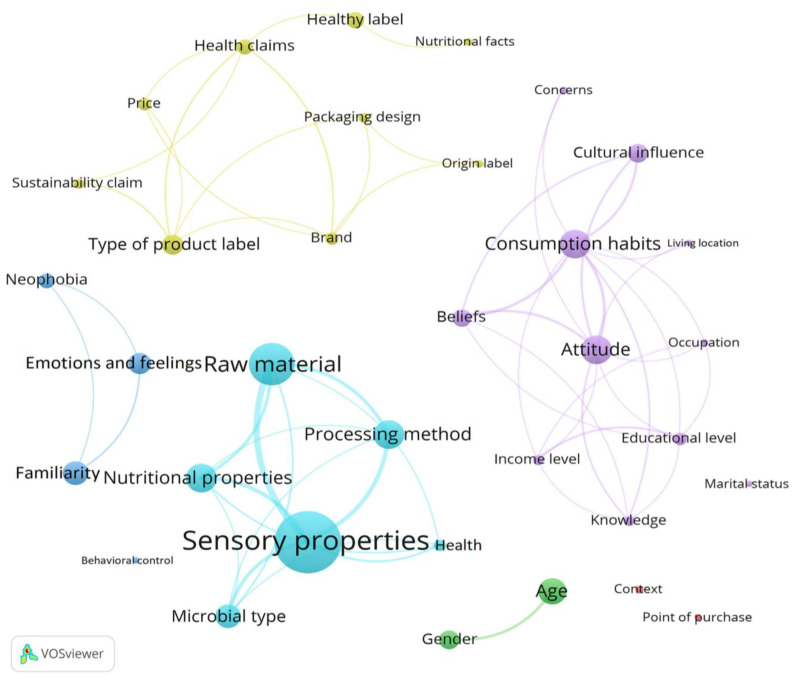
Map network analysis showing the associations among the sub-factors and the factors: cluster 1 situational factors (red), cluster 2 biological and physiological (green), cluster 3 psychological factors (blue), cluster 4 extrinsic product characteristics (yellow), cluster 5 sociocultural factors (purple), and cluster 6 intrinsic product characteristics (cyan).

A network analysis map was generated using the TXT files described in Section 2. The first TXT file contained information on the 34 sub-factors in Figure 3. Each sub-factor belongs to one of the six clusters given by the factors: situational factors, biological and physiological, psychological factors, extrinsic product characteristics, sociocultural factors, and intrinsic product characteristics. The sub-factors are presented by colored circles, where the larger the circle, the greater the frequency or weight. Meanwhile, the interactions occur through connection branches, where the wider the line, the greater its connection frequency. Figure 4 shows the results of the network analysis map, in this case representing the interaction between the sub-factors contained in each cluster. The most important sub-factor is sensory properties, mentioned at least 65 times in the reviewed articles, which belong to cluster 6. Sensory properties have a marked interconnection with raw materials, processing methods, and nutritional properties. As for the sociocultural factors of cluster 5, the most relevant sub-factors are attitude and consumption habits, followed by cultural influence and beliefs. This was the most complex network, with 11 sub-factors. Marital status was only mentioned once, so it did not interact with other sociocultural factors. Extrinsic product characteristics contained nine sub-factors, where the type of product label was the most relevant sub-factor, followed by the healthy label and health claims. Most of the sub-factors in this category were interconnected, although nutritional facts only interacted with healthy labels. Familiarity, along with emotions and feelings, were the most frequently mentioned variables in the psychological factor or cluster 3, followed by the neophobia sub-factor. It should be noted that behavioral control was not associated with the other psychological factors. The biological and physiological factors in cluster 2 only had the interconnected sub-factors age and gender. Interestingly, context and point of purchase belonging to cluster 1 situational factors do not interact, as they have not been studied together. The lack of interrelation between some sub-factors suggests that it would be necessary to analyze them together to understand the implication of this interaction on consumer behavior.

### 3.3. Analysis of Factors and Sub-Factors

The selected articles were sorted, categorized, and analyzed according to the six types of factors. The sub-factors are discussed in the following subsections.

#### 3.3.1. Biological and Physiological Factors

Within consumer perception and preference, biological factors such as age or gender play an important role [25,26]. In this sense, the perception of food changes with age [27]. Likewise, women tend to be more physiologically sensitive than men [28]. In the case of the FFPBs, it was found that age and gender are sub-factors that have been previously explored.

Several scientific reports have demonstrated the relationship between age and perception and preferences for fermented products [5,6,18,29,30,31]. The effect of age on consumer behavior towards FFPBs is influenced in different ways. Kwak et al. [32] and Rojas-Rivas et al. [33] state that the age of the consumer influences the perception of the image of a fermented product, especially in traditional products. On the other hand, Chezan and Antonelli [34] stated that even though young consumers may have a positive perception of a fermented product, it will not necessarily increase their purchase intention. Furthermore, age may be related to the amount of sugar added to natural yogurt, and the preference for products with certain attributes, especially in older adults [35,36]. Therefore, it is relevant to consider the age sub-factor as an issue to differentiate consumer segments or predict market evolution [32,37,38,39].

Participant gender is another sub-factor considered to drive perception and preferences for FFPBs. Gender influences perception and preferences related to presentation, appearance, and sensory characteristics of fermented products [36,39,40,41]. Women were the most sensitive, as they usually cook food [42]. In addition, women tend to be stricter with products containing artificial dyes [19]. On the contrary, men may be suitable targets for consuming different yogurt or honey-based fermented products [18,43,44]. Therefore, it is necessary to consider gender as a sub-factor that influences the use of products and/or the development of new products.

#### 3.3.2. Extrinsic Product Characteristics

Extrinsic characteristics are related to external features of the product, such as price, brand, packaging, label, and claims. When consumers are unaware of sensory properties, for example, when purchasing a new product, extrinsic characteristics are the main drivers of purchase intention creating the expectation about sensory characteristics [45]. Some extrinsic characteristics sub-factors are *brand, health, health claims, sustainability claims, healthy label, nutritional facts, origin label, origin, packaging design, price,* and *type of product label*.

The *brand* is a relevant sub-factor that impacts FFPB consumer behavior [46,47,48]; it guarantees a promise or contract with the manufacturer that results in a symbol of quality [49]. According to Arora et al. [40], brand recognition can be related to aspects allowing product differentiation. This is reflected in the purchase intention, especially local brands [29,46]. FFPB consumers trust recognized national brands and relate them to food safety and health [29].

*Health claims* provide information about the benefits of consuming one or more specific ingredients. This information must appear on the product’s label and may be related to positive effects. Therefore, this sub-factor has been studied in FFPB products, mainly in functional foods. Conti-Silva and Souza-Borgues [50] stated that health claims are essential to understand acceptance of functional food such as fermented milk. In the context of FFPBs, health claims positively influence consumers’ attention in food choice and consumption [18,48,50,51]. However, this effect is smaller than the sensory characteristics of products. Health claims for plant-based yogurt failed to increase liking levels by more than seven points due to the low sensory quality of this type of product [51]. Jaeger et al. [51] and Pinto et al. [48] concluded the same; this effect only occurs when the product has an adequate sensory quality.

Consumers can trust and make better decisions based on product information; therefore, health claims should be supported by up-to-date scientific studies [18,52]. The way claims are displayed is relevant, as a lack of clarity or poor visibility can impair consumers’ attention to differentiate between regular and healthier products [50,52]. Health claims must therefore be displayed clearly and objectively, as the type of language, amount of information, size of letter, size of framing, or the place in the package can affect the understanding of the information. Another relevant question is whether the consumer pays attention to the information provided. In this sense, Jaeger et al. [51] propose applying a self-report survey inquiring about the degree of attention to information or using eye tracking as an additional measure, although none of these tests can ensure that consumers pay attention.

The *healthy labels* sub-factor refers to additional product information, i.e., ingredients with health benefits, such as probiotics, antioxidants, sweeteners, protein levels, fiber, and salt reduction [11,37,41]. Many dairy products like yogurt and fermented milk contain probiotic strains that consumers associate with “healthy”, “aid digestion”, and “intestinal regulator” [5], and on the other hand, probiotics are associated with “healthy”, “protected”, “cheerful”, “yeast”, “microorganism”, “bacteria”, and “*Lactobacillus*” [52]. Consumers tend to associate health benefits with the type of product and not with specific ingredients such as probiotics. In this regard, Ávila et al. [5] pointed out that the label “probiotic” may cause disbelief or suspicion. The lack of a concrete opinion about a yogurt or fermented dairy product means that the information on the label does not influence the purchase intention. This could be due to a lack of understanding of the meaning of the concept “probiotic” and/or a lack of consumption of these products [5]. On the other hand, labels containing antioxidants could increase the intention to purchase the product. This is the case of the dry-fermented sausage, Cinta senese [53]. Sometimes information does not meet consumers’ expectations when the product is unacceptable [54]. Therefore, when designing new products, the nutritional value of food should be aligned with the sensory characteristics so that the consumer can perceive the health benefits, rather than focusing on negative aspects [41].

Nutritional facts were another sub-factor that refers to the government-regulated information on labels. It is presented as nutritional values, especially energy value, fat, saturated fat, sugar, and salt [55]. In plant-based fermented products, nutritional facts influence preference and willingness to pay for the products [12]. In addition, protein concentration can help increase the level of preference for these products [11].

Origin labeling is another sub-factor addressed in the study of FFPB perception and preference. Consumers prefer, appreciate, and trust local products [29,38], as they are familiar with the flavors and textures. Furthermore, freshness and health benefits could be linked to the product when the consumer is familiar with the raw material. The type of processing method could be associated with the country of origin, which is the case for Chinese products that use artificial processing, technology, and innovation [29].

Packaging design dictates how the consumer acquires information about a product, including its size, material, color, and image. It can also capture consumer’s attention and influence their expectations.

Like other products, FFPB packaging design influenced consumer response, i.e., the small size of drinkable yogurt is preferred because consumers perceive it as fresh, as shelf life is not compromised [29]. Packaging images may influence sensory expectations that affect willingness to purchase yogurt. Rebollar et al. [56] found that packaging with sugar images were perceived as less sweet, while sugar cubes were perceived as sweeter. In contrast, images of sugar bags generated an expectation of naturalness. Product images provide relevant information that can be easily processed and immediately capture the consumer’s attention. An image can generate different expectations, such as the level of sweetness and the naturalness of the ingredients used. According to Farah et al. [57], in some FFPBs, the shapes, colors, and images of the packaging appear confusing, i.e., different brands have similar packaging for a product category, making it difficult to differentiate between yogurt, fermented milk, and whey-based beverages. Appropriate packaging enables differentiation of FFPBs and conveys important information about the benefits and origins of these products.

Price is an important extrinsic sub-factor in the perception and preference of any food [45], as it is often related to product quality [6,46,58]. The willingness to purchase expensive FFPBs depends on consumer characteristics such as country of origin [59]. However, consumers increased their intention to pay premium costs when a product offers health benefits or health claims are provided in functional yogurt [18,59].

Sustainability claims refer to information about sustainability. Although some products are not labeled as sustainable, they are perceived as such by consumers due to their origin, i.e., the alternative fungal protein to meat is obtained from the fermentation of *Fusarium venenatum*, which consumers claim is environmentally sound and socially beneficial [34]. Furthermore, Greis et al. [31] observed that consumers consider plant-based yogurts to be sustainable, which affects product preference. In contrast, plant-based products might suffer from sensory acceptance regardless of marketing efforts to encourage consumption.

The type of product label is critical to differentiate products, especially within the same category. The way information is organized is relevant [47], particularly to differentiate a conventional product from a traditional one [60]. Kwak et al. [32] found that the “traditional” label did not affect acceptance, except in older adults, perhaps because they are more familiar with it and prefer traditional foods. The importance of labels for different consumer segments needs to be considered. Furthermore, the effect of the type of product label is related to personality traits and consumer attitudes [6,19]. In addition, product information can influence consumer expectations and perceptions [31,51,61]. While it is true that product information is presented in a relatively summarized form, it is necessary to consider that consumers with different levels of health consciousness may refute or corroborate this claim [62]. Therefore, consumers should be encouraged to read the general information on products to differentiate between them, i.e., nutritional facts [57].

#### 3.3.3. Intrinsic Product Characteristics

At the composition level, like any food, FFPBs represent a complex matrix composed of different macromolecules such as carbohydrates, lipids, proteins, vitamins, and water [13,63]. Additionally, the manufacturing process and raw material, among other variables, are responsible for the intrinsic characteristics [7,8,64,65]. Therefore, these attributes cannot be modified without altering the nature of the product and the perception that consumers have about food.

Among the intrinsic characteristics, the health-promoting effect sub-factor was found, which can be related to the following health promoters: pulque [33], fermented dairy products [5,52], and fungal protein-based or mycoprotein [34]. This coincides with the reports indicating that FFPBs provide health benefits [66]. However, it is important to consider the consumer’s perspective. Chezan et al. [34] stated that consumers with the highest score perceive fungal protein as less healthy and natural, indicating that although foods may be scientifically proven beneficial to health, it is important to understand consumers’ perception and attitudes toward products. This information may be useful to encourage the consumption of this type of product.

Fermentation is a chemical transformation process of complex compounds into simpler ones carried out by the action of different microorganisms such as bacteria, molds, and yeast [4,67,68]. Therefore, a key element in FFPBs is the microbial type, which is essential for sensory characteristics, consumer preferences, and health benefits [5,50,65,69]. The use of alternative starter cultures, such as *Lactobacillus* species, has been reported to improve sensory properties of FFPBs. In this sense, *L. plantarum*, *L. rhamnosus*, *L. casei*, *L. brevis*, and *Pediococcus pentosaccus* were used in the production of watermelon juice, where the preferred juices were those fermented with the last two species [10]. Alternative yogurt made from isolated lentil protein was fermented with *L. paracasei* FST 6.1, showing high acceptance, like the product obtained with the regular starter culture S. thermophiles. The sensory characteristics were less bitter aftertaste, with a more yogurt-like taste than the other strain [70]. In the case of fermented soybeans, five local Indonesian strains of the genus *Rhizopus sp*. were used, where the product fermented with *R. oligosporus* was observed to be the most preferable [71]. Interestingly, Cha et al. [72] proposed a fermentation process for edible insects, such as *P. brevitarsis* larvae, to decrease off-odors and improve taste. On the other hand, when *P. brevitarsis* was fermented with *S. cerevisiae* and GNIA 2, consumer preference was slightly increased, as volatile compounds perceived as chocolate aromas were generated.

The presence of lactic acid bacteria (LAB) has been reported in the production of FFPBs, mainly in some dry fermented sausages. Tukel and Sengun [73] and Pavli et al. [74] recommended the use of *L. plantarum* and *L. rhamnosus*, since fermented products using these strains improved the sensory characteristics resulting in increased preference. Coehlo et al. [75] reported that the taste of salami can be improved by using the probiotic *L. paracasei* LPC02. Although in many cases commercial strains were used, it is a fact that various strains of FFPBs were isolated, i.e., Nematí et al. [76] isolated seven LAB strains of the traditional fermented vegetable called “Toshi”, to develop a functional probiotic yogurt. The use of the unusual strains *S. thermophiles* and *L. bulgaricus* with the combination of *L. sakei* CJLS03 and *L. plantarum* C182 resulted in the most liked yogurt due to the probiotic properties. Then, the addition of probiotic strains and the enzymatic activity had a positive influence on the sensory characteristics of FFPBs. Moreover, it was possible to obtain odor and flavor profiles different from those of the starter culture alone, which contributed to consumer acceptance [73].

Sometimes, additional hedonic tests are necessary; da Cruz et al. [77] stated that fermented milk with *Bifidobacterium*, *Lactobacillus delbrueckii* subsp. *Lactis*, and *Lactobacillus casei* showed higher levels of preference. However, when the emotional response was evaluated, the product fermented with *Lactobacillus delbrueckii* subsp. *Lactis* had a higher citation of positive emojis, reflecting that further testing would be useful to understand consumer response. It is necessary to characterize the impact of the strains on fermentation, obtaining different alternatives in the elaboration of FFPBs.

Within intrinsic factors, nutritional properties are another sub-factor that is considered as a criterion that drives consumer perception and preferences and is based on the perceived image of probiotic content, low-fat, low calories, or reduced salt concentration [78]. The study showed that fermented products had a positive image due to their nutritional properties [5,48,52,67]. They are emerging as an alternative to prevent the global prevalence of disease such as obesity, diabetes, cardiovascular diseases, and lactose intolerance [34,79]. Consumers try to increase consumption of certain products if fat and salt content has been reduced [8,37].

Sugar content has been explored in fermented dairy products. According to Torrico et al. [79] and De Souza et al. [62], in yogurt products, the sugar concentration can be reduced by up to 40%. However, when the consumer has the freedom to add the amount of sugar, they often pour more sugar than the commercial one [35]. Additionally, the use of natural sweeteners is an alternative to reduce sucrose in yogurts [80], and the type of sweetener impacts the final amount of added sugar in the yogurt [35]. On the other hand, Esmerino et al. [52] found that consumers perceive probiotic yogurt and fermented milk with probiotics as nutritious, while low-fat fermented milk is perceived as low in calories when it is part of the diet. The concepts light, zero fat, zero lactose, and sugar-free were associated with dietary restriction and loss of sensory quality, especially by Brazilian consumers [58].

Preference for yogurt based on its fat content depends on the consumer’s country of origin [59], i.e., Polish students preferred regular yogurt while Taiwanese liked fatty yogurts. Janiaski et al. [81] reported that skimmed yogurts were less preferred than low-fat yogurts. Rutkowska et al. [36] observed that lactose-free kefir consumed by lactose-intolerant elderly people was more appreciated than regular kefir because it was perceived as sweeter and milkier. In contrast, products formulated with higher concentrations of health-promoting ingredients such as agar or insoluble triticale (wheat or oat) were not preferred by consumers, even when the product provided a source of dietary fiber [63,82]. Plant-based yogurts are associated with nutritional properties due to their protein content, which influences preferences [12,13]. Therefore, nutritional properties should be considered to encourage the consumption of FFPBs.

Another intrinsic sub-factor found in the review was the processing method that can affect the perception of the product. For traditional products, the processing method is critical to differentiate the variability of the product, mainly due to the artisanal process [60,83,84], which influences preferences [7,30,32,38,53,85,86,87], in addition to the perception of product image [9]. Information provided to the consumer about the type of process is another relevant sub-factor, especially when such information is decisive in improving the preference for the evaluated products among millennial consumers [38]. Various studies have been conducted to optimize the production process of non-alcoholic beverages based on fermented rice [88] and kombucha [89]. This has improved the acceptability of the product, especially when some off-flavors or unpleasant sensations are obtained in the common process. Sikombe et al. [86] mentioned the need to improve production processes and product quality, particularly when it comes to traditional products such as Mabisi, as consumers are now more aware of their food environment.

As for innovations in new product development, the processing method can influence the perception and preferences of FFPBs [65]. According to Hay et al. [29], the use of different technologies in the processing method for product development could have a moderate effect on consumer perception. Similarly, different fermented processing methods were applied to an indigenous vegetable to minimize postharvest loss, which improved sensory quality and overall acceptability [90].

Additionally, the processing method is crucial in consumer preferences when looking to add value to by-products such as whey [91] or orange peels [65]. It is also necessary to consider that the processing method should be as minimal as possible, since a minimally processed product is perceived as having higher value [92].

Among the intrinsic factors, the raw material used in FFPBs influences consumer perception and preference. Raw materials are vital, especially in traditional products, as they contribute to the identity of a product associated with a geographical region [38,83,85,93,94]. In some cases, within the same region, products considered traditional may have different characteristics. Byeon et al. [95] pointed out that the processing method may influence these characteristics. Similar results were obtained for Douchi, a traditional Chinese condiment [87], and Attiéké, an Ivorian side dish [96]; in both cases, the sensory characteristics and aromatic profile were associated with the combination of the raw material and the production process.

Different studies have been conducted on meat products to analyze the effect of raw materials on the acceptance of fermented products. One challenge is to estimate the proportion of raw material without compromising the level of liking [7,39,62,81,89,97]. Therefore, knowledge of the effects of raw materials on acceptability can be a starting point to reduce potential negative effects [7,54]. According to Shan et al. [8], the raw materials used in the production of fermented meat products should be compatible with each other, as this may influence the acceptance of the products. The type of raw material and the processing methods are crucial for the acceptance of salamis, since both affect the color, i.e., too dark or light a color reduces acceptability [98]. Furthermore, replacing fat with different raw materials [54] and exploring different meat species such as donkey could also have an impact [99].

Furthermore, the use of different raw materials in the development of new products is important for the diversification of FFPBs [50,64,88,90] and for creating sustainable food, for example, using *Aspergillus oryzae* in vegetarian burger patties [100]. It is necessary to consider that consumers tend to look for products containing natural raw materials [6,101,102]. As for fermented dairy products, it has been observed that plant-based products are an alternative source of protein dairy products. Combining raw materials can be a technological challenge for the food industry [13,31], for underutilized raw materials such as whey [81,91]. Saint-Eve et al. [12] mentioned that fermented foods that combine animal and vegetable raw materials can be a suitable alternative to design more sustainable diets, since consumers have a positive perception of these products. Furthermore, it is necessary to consider that the nature and quality of raw materials also influence consumer behavior [5,6], especially in the case of probiotic products such as kombucha, where the use of different types of teas has been explored [103]. The raw material used to fortify FFPBs also affects consumer preference; for example, yogurt fortified with insoluble fiber from different sources [82] and fermented fortified cereal based in non-alcoholic beverages with legumes [104].

The 9-point hedonic scale is the most widely used methodology to determine the level of preference for a product; however, a better understanding will be obtained if other variables such as liking emotions and physiological response are measured. Gupta et al. [105] used different types of yogurts to measure these responses. For this purpose, different videos were recorded during the test sessions, while the consumer’s heart rate, facial expressions, and emotional responses were measured.

According to the studies analyzed, sensory properties represent one of the most important drivers of consumer perception and preference for FFPBs. However, the type of sensory properties that influence the level of liking may change, as it depends on the type of product [57,64,97,106,107]. In the case of fermented dairy products, sensory properties can influence preferences positively and negatively [50,52]. The influence of sensory properties is associated with the origin of the population, the residence time [108], and the population age [31,59]. In the development of new products, optimization, or adaptation of FFPBs, sensory properties are essential [11,62,63,65,72,74,75,82,91], especially when it is necessary to differentiate them [43,48,57,67,103,109]. Sometimes it is necessary to add flavoring to improve the taste, affecting the liking level [36,80,81,102,109]. Mandha et al. [10] mentioned that the descriptors considered as “natural characteristics” are more appreciated; on the contrary, descriptors of sour, bitter, and aftertaste are less appreciated characteristics. The characterization of sensory properties is an important topic in the FFPB industry, as it provides information for developing products that meet consumer expectations. Plant-based yogurts compared to dairy yogurts have a lower level of liking due to poor sensory quality [51]. However, in some cases, it has been found that characteristics like those of dairy yogurts, such as white color, smooth appearance, sweet taste, and texture, could have a positive influence on the preference for plant-based yogurts [13,31,70,106,110,111]. In the case of fermented plant-based beverages, color, smell, and taste influenced the purchase intention of the product [88,110]. In contrast, products based on fungal proteins have a neutral taste, which should be considered a point of improvement, since consumers expected a better taste and texture [34,100,101,112]. In the case of dry-cured meat products, sensory properties such as flavor, appearance, and texture are the most important factors affecting purchase and consumption [39,54,99]. As for salami, the sensory properties that predict the liking level are related to the spicy flavor. In the specific case of Napoli salami, it is the pepper flavor, and a ripened odor [7] for *Cacciatore* salami, with the main characteristics being spicy pepper and fennel seeds [39]. In the case of appearance characteristics, color is the most important sensory characteristic. According to different studies, the dimensions of the fat pieces and the ability to melt on the palate are determining factors for the consumer [7,73,93,99], so tenderness affects the overall acceptability [98]. In traditional products, sensory properties are important in product preference and identity [42,60,83,84,113]. Furthermore, when unexpected or different flavors are found in traditional products, the acceptance might decrease [114]. Sensory properties might be related to the way a traditional product is consumed [30,32,33,71,94], which is relevant for the identification of liking drivers [30,86,87,95,96]. Likewise, the identification of the sensory properties responsible for preferences in traditional products is a fundamental issue when it is desired to adapt them to different types of consumers [37,38,46,83,85]. However, it is necessary to consider to what extent such adaptations can be made, since they could influence the consumer’s perception of the product image [115].

#### 3.3.4. Psychological Factors

The role played by psychological factors in food choice is documented in Refs. [20,116]. Within the psychological factor, various sub-factors were found, including perceived behavioral control, which reflects the perceived ease or difficulty of performing a behavior, and is related to self-efficacy [117]. According to Mustapa et al. [110], perceived behavioral control is a crucial predictor of purchase intention for plant-based fermented products. That is, consumers trust that the products are readily available and therefore can easily purchase them without facing difficulties. Emotions and feelings were another sub-factor related to the psychological area. In addition to sensory sub-factors, it is important to understand that consumption is a complex experience that involves several factors where emotions and feelings are part of this process [51,103,105].

Measuring this sub-factor is important to differentiate products with similar levels of liking [19,48,52,77]. Emotions could better explain the level of liking [13]; this may be due to the relationship between emotions and sensory characteristics, since positive emotions are generated that contribute to high arousal [111]. Jaeger et al. [106] pointed out specific links between sensory drivers and emotional aspects generated by the products. In this line, Fibri and Frøst [38] mention that when it comes to traditional fermented food, different emotions are generated, particularly pride. In addition, by measuring emotions and feelings, it is possible to obtain complementary information for the development of new products, packaging and labels, marketing, and commercialization strategies [13,48,77,106].

Within the choice and consumption of FFPBs, familiarity has been reported, especially in functional products, since the degree of familiarity of the consumer influences the interpretation of the information and judgments about the quality of the product [6]. Similarly, familiarity influences the perception of the sensory characteristics of the product, since the recognition of these characteristics allows the consumer to identify and differentiate between diverse categories of fermented products [10,18,19,29,52,69,109], particularly when they are perceived as traditional products [7,30,115]. Furthermore, consumer familiarity with products having similar characteristics to new alternatives tends to dissipate consumer concerns about alternative products [34]. Therefore, when developing new FFPBs, consumer familiarity should be considered [94].

On the other hand, unlike familiarity, neophobia is a psychological sub-factor that refers to the reluctance to try new or unfamiliar foods [9,31,118], making it an important aspect of eating behavior. According to Bernal-Gil et al. [9], the level of neophobia is related to the perception and intention to try an ethnic fermented beverage, since high levels of neophobia are related to the perception of negative characteristics. Similarly, high levels of neophobia influence the perception of unfamiliar products, as they are considered unsafe, unhealthy, and unnatural [34]. This may be because the level of neophobia is related to the level of memory of food characteristics and experiences [38]. A possible alternative to counteract the effects of neophobia is to inform consumers about product characteristics [43,61]. Therefore, understanding the characteristics of neophilic and neophobic consumers is fundamental in marketing strategies [34].

#### 3.3.5. Situational Factors

The systematic review identified the situational factors, within which are the sub-factors context and point of purchase. It is important to consider that food choices are made in a specific situation that influences decisions, i.e., in a specific context. In this sense, context refers to the specific situational and temporal conditions under which products are chosen or consumed [119,120]. Saint-Eve et al. [35] pointed out that the evaluation of consumer behavior within a given context provides information as close to reality as possible. In this sense, the type of consumption and perception of yogurt is related to the context in which it is consumed. It can be considered as part of breakfast, a mid-morning snack, or a dessert or drink after dinner or before bed [13]. The type of product, habits, and culture must be also considered.

On the other hand, the point of purchase can influence the perception of the image and quality of a fermented product. Rojas-Rivas et al. [33] reported that consumers consider *pulquerías* (a place where pulque is sold) to be dirty and harmful places, which is related to a negative reputation of the beverage. Therefore, the owners of the point of purchase must improve these spaces to not affect the perception of consumers.

#### 3.3.6. Sociocultural Factors

Sociocultural factors play a special role in food choice and consumption [121], and FFPBs are no exception. In sociocultural factors, the attitudes are documented in FFPBs. In the food choice process, attitude is the result of an individual’s positive or negative evaluation of the outcome of this process. The resulting attitude is based on the consequences of the behavior and the value the individual places on those consequences [110,111]. Thus, attitudes can positively or negatively affect the perception and choice of fermented products [5,19,48,52,94,106]. Coincidentally, Jaeger et al. [106] reports that when a negative attitude is shown, there is less willingness to pay for plant-based yogurts. Likewise, a negative attitude is related to the name of the product, such as fungi-based foods, due in part to their ability to spoil food [34,101]. On the other hand, product image can influence consumer attitudes [56]. When it comes to ethnic products, attitude measurement helps to identify perception towards this type of product [9]. Additionally, attitude assessment is useful for identifying consumer segments [31,35,38]. Therefore, to avoid negative consumer attitudes, it is necessary to develop information strategies aimed at increasing consumer awareness of the benefits of fermented products [40,61].

The beliefs that consumers may have about food or food groups are, in most cases, built from cultural and social aspects [20,122]. The influences of beliefs within FFPBs on consumer perceptions have been demonstrated [54,61], since in traditional products, beliefs influence the perception of these products in different ways. Rojas-Rivas et al. [33] point out that pulque consumers consider that this product has health benefits that promotes breastfeeding, which has been maintained since pre-Hispanic times. According to Bernal-Gil et al. [9], in traditional products, beliefs about the manufacturing process and ingredients are the most important characteristics for the perception of the image of traditional products. On the other hand, Pinto et al. [92] mention that in fermented dairy products, the reputation of health benefits generates positive beliefs. According to Hay et al. [29], the beliefs about the sensory characteristics that a product should have were determinants for its choice. Furthermore, Mustapa et al. [110] mention that consumers’ belief in purchasing power plays a crucial role in shaping purchase intentions. On the contrary, while it is true that consumers believe that fermented dairy products provide health benefits, they think that the products have specific target groups, and marketing does not encourage another market niche [52]. For this reason, it would be necessary to include other consumer segments to encourage the consumption of fermented products.

Another sub-factor studied in the context of FFPBs was concerns. According to Penna et al. [19], sustainable, healthy alternatives have increased consumer concerns. In this regard, do Carmo et al. [58] point out that due to the COVID-19 pandemic, a segment of consumers showed an increase in health concerns, influencing the purchase intention of yogurt. Consumers with this behavior expect to get pleasure from these products. On the contrary, the decrease in health concerns would be related to dietary restriction. This may have generated an overload of stress, making food considered as an escape and favoring excessive consumption, especially of “comfort foods”. Considering that the concerns reflect different factors mainly of sociocultural nature [20], it is necessary to expand the study of this sub-factor, especially in fermented products considered alternative food.

Another sociocultural sub-factor is consumption habits, which are stable over time [123] and are part of the determinants of eating behavior, since they contribute to the learning and developing of food preferences [124]. In the context of FFPBs, the factors that contribute to the formation of consumption habits are multidimensional and are related to sensory and no-sensory characteristics, including psychological aspects (emotions, feelings, personality traits), preparation methods, and communication (marketing) [9,52,57,97]. The effect of consumption habits on preferences is based on socio-demographic characteristics such as age, gender, educational level, having children, and region of residence [5,29,108], together with repeated exposure to the product [69]. FFPB consumption habits are linked to treatment and improvement of health and certain dietary restrictions [77,92]. Consumption habits guide the appropriate time to consume a product such as yogurt, including the amount of sugar added to the product [35,52,58]. In addition, consumption habits influence awareness and perception of fermented products [40,44,98]. Regarding the consumption of meat alternatives, Chezan et al. [34] point out that products based on fungal proteins are consumed by those who tend to reduce meat consumption. Considering the previous information, the study of consumption habits is important to develop high-quality products, influencing the perception of fermented products, and therefore encouraging their consumption [59].

Within each culture, categories, norms, and values often regulate what is considered acceptable food, the proportion or quantity of food, the combination, and the context in which the food should be consumed [125]. Evidence of this cultural influence is provided by the differences in the level of liking for FFPB with sensory characteristics according to cultural expectations, intrinsic attributes, and place of residence [29,31,85,94]. Hay et al. [29] mention that cultural influence can be reflected in consumers’ perception of yogurt reliability and product shelf life, especially if the transport time is long. When consumers are exposed to a culture, food choices may change. According to de Matos et al. [108], this process can take at least three years. However, Banovic and Grunert [61] mention that when relatively unknown fermentation technologies are evaluated at a conceptual level, no cultural differences are observed. Because of this situation, Park [115] suggested that the mechanisms underlying the cultural factor should be studied. The information obtained would be useful for developing products adapted to consumers from different regions [59,86].

According to the information reviewed, education level represents a sub-factor that affects the perception and preference of FFPBs [40,43]. The influence of education level may be due to consumers being more critical in terms of quality [41]. Furthermore, when it comes to the revaluation of traditional fermented products, consumers with higher education levels tend to be more sensitive [33,83].

Among the sociocultural factors, income level is a determining factor in consumer preferences [20]. In this sense, different studies confirm that income influences the perception and preferences of FFPBs, as mentioned by Arora et al. [39]. De Devitiis et al. [43] reported that consumers with high income levels are more likely to accept, prefer, and consume healthy and sustainable foods such as yogurt based on sheep’s milk. In addition, the assessment of the health benefits and naturalness of products such as yogurt depends on different factors, among which the income level is the most important [41]. However, despite its importance, in the context of FFPBs, the income level has been scarcely studied.

Knowledge is a sub-factor used as a moderator in consumer choice of FFPBs [40]. According to Esmerino et al. [52], consumer knowledge tends to influence the purchase intention of fermented dairy products. The level of knowledge about the fermentation process can influence the perception of the product. Deba-Rementeria et al. [65] mentioned that Spanish consumers know that fermentation processes improve sensory properties preserving foods. Therefore, communication strategies should be designed to emphasize the benefits obtained from FFPB consumption, especially in the fermentation process.

Finally, living location [34], marital status [86], and occupation [40] were identified as sub-factors influencing the perception and preferences of fermented products. It is important to mention that only one article per sub-factor was identified. Therefore, it would be necessary to consider these sub-factors as relevant to be studied in fermented foods.

### 3.4. Implications for the Fermented Food and Beverage Category

The potential of fermented food products and beverages as sustainable alternative food sources with health benefits has mostly contributed positively to consumer expectations. The articles analyzed in this review show that intrinsic attributes, particularly sensory attributes, are the most important sub-factors influencing consumer behavior. On the other hand, factors related to biological and physiological aspects, along with situational factors, have been the least studied. Due to this situation, it would be necessary to carry out consumption and preference studies in different contexts, especially in real situations, since this aspect has been scarcely studied in FFPBs. It is therefore important to note that the process of food choice is complex. A reflection of this is that while extrinsic factors may improve acceptability, this may occur only when the sensory quality of the FFPB is adequate.

FFPB products that have been widely studied are yogurt and fermented milk; however, according to the review, development and innovation in FFPBs have grown, focusing on sustainable and nutritious foods. These developments have been aimed at revaluing by-products such as whey, at innovating fermentation processes to increase the shelf life of products, or at improving the flavor of those that by their nature are still little accepted, as in the case of plant-based products. Some products that are poorly known or rejected by consumers due to their origin have been studied, i.e., products derived from insects or alternative protein sources such as mushroom-based products. It would be advisable to carry out an expanded study of factors that have been scarcely studied. These factors could include neophobia and emotions, as well as the evaluation of communication strategies to familiarize consumers with these products and understand what benefits can be obtained from their consumption

One aspect that has been sought to be highlighted within FFPB has been the “sustainability claim”. Consumers perceive FFPBs as sustainable, especially those of plant origin and those based on fungi. However, it is necessary to analyze, from a holistic approach, the impact that other types of information can have on consumer behavior. Hellwing et al. [112] analyzed the effect of additional information such as the production process, processing costs, and aspects related to environmental benefits, i.e., the environmental awareness that consumers may have. This sub-factor would be related to the level of awareness that consumers may have about the impact that the production of this type of food has on the environment. Fibri and Frøst [38] indicate that providing consumers with information about raw materials, processes, and ecological measures can influence consumers’ understanding of FFPBs. This may encourage the consumption of these products, since lack of knowledge can be a barrier to their consumption, especially if they are alternative products [101].

Likewise, Greis et al. [31] proposed to conduct studies on consumer characteristics such as personality, emotions, gender, and age groups to provide alternatives appropriate to the consumer profile aimed at sustainable food. While it is true that the level of liking tests are measures that allow estimating the hedonic response of consumers, they cannot reflect the totality of consumer behavior. Therefore, it is necessary to apply different methodologies and tools that relate the hedonic response to physiological measures such as heart rate, or psychological aspects such as emotions or personality traits, as proposed by Gupta et al. [105] and Jaeger et al. [106].

Finally, it would be advisable to adopt a multidisciplinary approach, which would allow addressing the different factors in a comprehensive manner, which would allow having a broader perspective of both the products and the consumers, allowing a better understanding of the aspects that influence consumer behavior and making improvements to the product for its acceptance and consumption [42,43].

### 3.5. Strengths and Limitations of the Review

Although this study proposes a multidisciplinary approach to analyze the factors that influence the consumption of fermented foods and beverages, it does not consider the number of publications per region. This information could be essential to encourage the study of the factors that influence behavior, to promote the consumption of FFPBs. In this sense, scientific articles not published in English were not considered, so the lack of inclusion of these papers could be a bias. These works could have considered local or regional FFPB as an important part of the gastronomic diversity of these regions.

## 4. Conclusions

As a result of the systematic review, 92 articles were found that studied different drivers of perception and preference for FFPBs. Inspired by the model proposed by Mojet [21], it was possible to identify and categorize the consumers’ drivers of perception and preference for FFPBs. Through the model, it was possible to identify six different factors: (1) biological and physiological, (2) extrinsic product characteristics, (3) intrinsic product characteristics, (4) psychological factors, (5) situational factors, and (6) sociocultural factors. Considering the number of articles, the intrinsic product characteristics were the most studied factor, particularly the sensory ones. This suggests that within the FFPBs, the sensory part is the most important driver of consumer behavior. In addition, the network analysis was useful in showing the lack of relationship between some sub-factors, so it would be convenient to analyze these sub-factors together as far as possible. On the other hand, the situational factor was the least studied; therefore, it would be necessary to consider extending the study to understand its role in FFPBs. In addition, since FFPBs may represent an alternative food with health benefits, it would be necessary to consider expanding the variety of fermented foods, mainly those that have been scarcely studied, since most of the studies have focused on yogurt. This could contribute to the diversification of fermented product alternatives. Likewise, future perspectives will adopt multidisciplinary approaches to understand the role of the different factors studied. This would allow the development of new products that meet the needs of consumers and, in addition, enable the development of thoughtful markets.

## Data Availability

No new data were created or analyzed in this study.

## Supplementary Materials

The following supporting information can be downloaded at: https://www.mdpi.com/article/10.3390/foods14050713/s1, Tables S1–S6: Articles identified in the systematic search and the factors and sub-factors studied. Table S7: Comparison of the proportions of studies that addressed the different factors according to Mojet’s model [21]. Tables S8–S13: Comparison of the proportions of studies that addressed the different sub-factors according to Mojet’s model [21].
