# Supplementary material for: Consumers’ Drivers of Perception and Preference of Fermented Food Products and Beverages: A Systematic Review
Source: Foods. 2025 Feb 20;14(5):713. doi: 10.3390/foods14050713 (PMC11899150; doi:10.3390/foods14050713)
Supplement: Supplementary file 1 [file foods-14-00713-s001.zip › foods-3424102-supplementary.pdf]

Table S1. Articles identified in the systematic search and the factors and sub-factors studied.

Author	Factor	Sub factor
Adinsi et al., [83]	Intrinsic product characteristics	Processing method, raw material, sensory properties
	Sociocultural factors	Educational level
Akanni et al. [104]	Intrinsic product characteristics	Raw material
Akissoé et al., [85]	Intrinsic product characteristics	Processing method, raw material, sensory properties
	Sociocultural factors	Cultural influence
Ambarita et al., [114]	Intrinsic product characteristics	Sensory properties
Arora et al., [40]	Biological & Physiological	Gender
	Extrinsic product characteristics	Brand
	Sociocultural factors	Attitude, consumption habits, education level, income level, knowledge, occupation
Ávila et al., [5]	Biological & Physiological	Age
	Extrinsic product characteristics	Healthy label
	Intrinsic product characteristics	Health, microbial type, nutritional properties, raw material
	Sociocultural factors	Attitude, consumption habits
	Sociocultural factors	Consumption habits
Banovic and Grunert [61]	Extrinsic product characteristics	Type of product label
	Psychological factors	Neophobia
	Sociocultural factors	Attitude, beliefs, cultural influence
Bernal-Gil et al., [9]	Intrinsic product characteristics	Processing method
	Psychological factors	Neophobia
	Sociocultural factors	Attitude, beliefs, consumption habits
Boeck et al., [68]	Intrinsic product characteristics	Microbial type, sensory properties
Brückner-Gühmann et al. , [11]	Extrinsic product characteristics	Healthy label, nutritional facts
	Intrinsic product characteristics	Sensory properties
Burgess [46]	Extrinsic product characteristics	Brand, price
	Intrinsic product characteristics	Sensory properties
Byeon et al., [95]	Intrinsic product characteristics	Raw material, sensory properties
Cais-Sokolińska and Walkowiak-Tomczak [107]	Intrinsic product characteristics	Sensory properties
Cardello et al., [111]	Intrinsic product characteristics	Sensory properties
	Psychological factors	Emotions and feelings
	Sociocultural factors	Attitude
Cha et al., [72]	Intrinsic product characteristics	Microbial type, sensory properties
Chezan et al., [34]	Biological & Physiological	Age
	Extrinsic product characteristics	Sustainability Claim
	Intrinsic product characteristics	Health, sensory properties
	Psychological factors	Familiarity, neophobia
	Sociocultural factors	Attitude, consumption habits, living location

Table S2. Articles identified in the systematic search and the factors and sub-factors studied (Cont.)

Author	Factor	Sub factor
Chijioke et al., [42]	Biological & Physiological	Gender
	Intrinsic product characteristics	Sensory properties
Coelho et al., [75]	Intrinsic product characteristics	Microbial type, sensory properties
Conti-Silva and de Souza-Borges [50]	Extrinsic product characteristics	Health claims
	Intrinsic product characteristics	Microbial type, raw material, sensory properties
Costa et al., [69]	Intrinsic product characteristics	Microbial type
	Psychological factors	Familiarity
	Sociocultural factors	Consumption habits
Costa et al., [109]	Intrinsic product characteristics	Sensory properties
	Psychological factors	Familiarity
da Cruz et al., [75]	Intrinsic product characteristics	Microbial type
	Psychological factors	Emotions and feelings
	Sociocultural factors	Consumption habits
Dartora et al., [103]	Intrinsic product characteristics	Raw material, sensory properties
	Psychological factors	Emotions and feelings
De Devitiis et al., [43]	Biological & Physiological	Age, gender
	Intrinsic product characteristics	Sensory properties
	Psychological factors	Neophobia
	Sociocultural factors	Educational level, income level
de Matos et al., [108]	Intrinsic product characteristics	Sensory properties
	Sociocultural factors	Consumption habits, cultural influence
De Souza et al., [62]	Extrinsic product characteristics	Type of product label
	Intrinsic product characteristics	Nutritional properties, raw material, sensory properties
Deba-Rementeria et al. [65]	Intrinsic product characteristics	Microbial type, processing method, sensory properties
	Sociocultural factors	Knowledge
Di Monaco and Cavella [7]	Intrinsic product characteristics	Processing method, raw material, sensory properties
	Psychological factors	Familiarity
Dias et al., [67]	Intrinsic product characteristics	Nutritional properties, sensory properties
Diby et al., [97]	Intrinsic product characteristics	Raw material, sensory properties
do Carmo et al., [58]	Extrinsic product characteristics	Price
	Intrinsic product characteristics	Nutritional properties
	Sociocultural factors	Concerns, consumption habits
Eidt et al., [44]	Biological & Physiological	Gender
	Sociocultural factors	Consumption habits

Table S3. Articles identified in the systematic search and the factors and sub-factors studied (Cont.)

Author	Factor	Sub factor
Esmerino et al., [52]	Extrinsic product characteristics	Health claims, healthy label
	Intrinsic product characteristics	Health, nutritional properties, sensory properties
	Psychological factors	Emotions and feelings, familiarity
	Sociocultural factors	Attitude, beliefs, consumption habits, knowledge
Farah et al., [57]	Extrinsic product characteristics	Packaging design, type of product label
	Intrinsic product characteristics	Sensory properties
	Sociocultural factors	Consumption habits
Fibri and Frøst [38]	Biological & Physiological	Age
	Extrinsic product characteristics	Origin label
	Intrinsic product characteristics	Processing method, raw material, sensory properties
	Psychological factors	Emotions and feelings, neophobia
	Sociocultural factors	Attitude
Freschi et al., [39]	Biological & Physiological	Age, gender
	Intrinsic product characteristics	Raw material, sensory properties
Greis et al., [31]	Biological & Physiological	Age
	Extrinsic product characteristics	Sustainability claim, type of product label
	Intrinsic product characteristics	Raw material, sensory properties
	Psychological factors	Neophobia
	Sociocultural factors	Attitude, cultural influence
Gupta et al., [13]	Intrinsic product characteristics	Nutritional properties, raw material, sensory properties
	Psychological factors	Emotions and feelings
	Situational factors	Context
Gupta et al., [105]	Intrinsic product characteristics	Raw material
	Psychological factors	Emotions and feelings
Hay et al., [29]	Biological & Physiological	Age
	Extrinsic product characteristics	Brand, origin label, packaging design
	Intrinsic product characteristics	Processing method
	Psychological factors	Familiarity
	Sociocultural factors	Beliefs, consumption habits, cultural influence
Hellwig et al., [112]	Intrinsic product characteristics	Sensory properties
Hellwig et al., [101]	Intrinsic product characteristics	Raw material, sensory properties,
	Sociocultural factors	Attitude

Table S4. Articles identified in the systematic search and the factors and sub-factors studied (Cont.)

Author	Factor	Sub factor
Jaeger et al., [51]	Extrinsic product characteristics	Health claims, sustainability claim, type of product label
	Intrinsic product characteristics	Sensory properties
	Psychological factors	Emotions and feelings
	Sociocultural factors	Attitude
Jaeger et al., [106]	Intrinsic product characteristics	Sensory properties
	Psychological factors	Emotions and feelings
	Sociocultural factors	Attitude
Janiaski et al., [81]	Intrinsic product characteristics	Nutritional properties, raw material, sensory properties
Jo and Kim [84]	Intrinsic product characteristics	Processing method, sensory properties
Kim and Lee [60]	Extrinsic product characteristics	Type of product label
	Intrinsic product characteristics	Processing method
Kim and Lee [113]	Intrinsic product characteristics	Sensory properties
Kim et al., [94]	Intrinsic product characteristics	Raw material, sensory properties
	Psychological factors	Familiarity
	Sociocultural factors	Attitude, cultural influence
Kim et al., [98]	Intrinsic product characteristics	Raw material, sensory properties
Kwak et al., [32]	Biological & Physiological	Age
	Extrinsic product characteristics	Type of product label
	Intrinsic product characteristics	Processing method, sensory properties
Lee et al., [37]	Biological & Physiological	Age
	Extrinsic product characteristics	Healthy label
	Intrinsic product characteristics	Nutritional properties, sensory properties
Lücke et al., [64]	Intrinsic product characteristics	Raw material, sensory properties
Mandha et al., [10]	Intrinsic product characteristics	Microbial type, sensory properties
	Psychological factors	Familiarity
Mantilla et al., [63]	Intrinsic product characteristics	Nutritional properties, sensory properties
Marino et al., [99]	Intrinsic product characteristics	Raw material, sensory properties
Marino et al., [54]	Extrinsic product characteristics	Healthy label
	Intrinsic product characteristics	Raw material, sensory properties
	Sociocultural factors	Beliefs, consumption habits
Mishra et al., [88]	Intrinsic product characteristics	Processing method, raw material, sensory properties
Mustapa et al., [110]	Intrinsic product characteristics	Sensory properties
	Psychological factors	Behavioral control
	Sociocultural factors	Attitude, beliefs
Napolitano et al., [93]	Intrinsic product characteristics	Raw material, sensory properties
Nemati et al. [76]	Intrinsic product characteristics	Microbial type

Table S5. Articles identified in the systematic search and the factors and sub-factors studied (Cont.)

Author	Factor	Sub factor
Oliveira et al., [47]	Extrinsic product characteristics	Brand, health claims, type of product label
Oliveira et al., [102]	Intrinsic product characteristics	Raw material, sensory properties
Panjapiyakul et al., [96]	Intrinsic product characteristics	Raw material, sensory properties
	Sociocultural factors	Consumption habits
Park et al., [115]	Intrinsic product characteristics	Sensory properties
	Psychological factors	Familiarity
	Sociocultural factors	Cultural influence
Pavli et al., [74]	Intrinsic product characteristics	Microbial type, sensory properties
Penna et al., [6]	Biological & Physiological	Age, gender
	Extrinsic product characteristics	Price, type of product label
	Intrinsic product characteristics	Raw material
	Psychological factors	Familiarity
Penna et al., [19]	Extrinsic product characteristics	Type of product label
	Psychological factors	Emotions and feelings, familiarity
	Sociocultural factors	Attitude, concerns
Pereira et al., [80]	Intrinsic product characteristics	Nutritional properties, sensory properties
Pinto et al., [92]	Intrinsic product characteristics	Nutritional properties, processing method
	Sociocultural factors	Beliefs, consumption habits
Pinto et al., [48]	Extrinsic product characteristics	Brand, health claims
	Intrinsic product characteristics	Sensory properties
	Psychological factors	Emotions and feelings
	Sociocultural factors	Attitude
Rebollar et al., [56]	Extrinsic product characteristics	Packaging design
	Sociocultural factors	Attitude
Roh et al., [30]	Biological & Physiological	Age
	Intrinsic product characteristics	Processing method, sensory properties
	Psychological factors	Familiarity
Rojas-Rivas et al., [33]	Biological & Physiological	Age
	Intrinsic product characteristics	Health, sensory properties
	Situational factors	Point of purchase
	Sociocultural factors	Beliefs, educational level
Rousta et al., [100]	Intrinsic product characteristics	Raw material, sensory properties

Table S6. Articles identified in the systematic search and the factors and sub-factors studied (Cont.)

Author	Factor	Sub factor
Rutkowska et al., [36]	Biological & Physiological	Age, gender
	Intrinsic product characteristics	Nutritional properties, sensory properties
Saint-Eve et al., [35]	Biological & Physiological	Age
	Intrinsic product characteristics	Nutritional properties
	Situational factors	Context
	Sociocultural factors	Attitude, consumption habits
Saint-Eve et al., [12]	Extrinsic product characteristics	Nutritional facts
	Intrinsic product characteristics	Nutritional properties, raw material
Sajdakowska et al., [41]	Biological & Physiological	Gender
	Extrinsic product characteristics	Healthy label
	Sociocultural factors	Educational level, income level
Sangija et al., [90]	Intrinsic product characteristics	Processing method, raw material
Shan et al., [8]	Intrinsic product characteristics	Nutritional properties, raw material
Sikombe et al., [86]	Intrinsic product characteristics	Processing method, sensory properties
	Sociocultural factors	Cultural influence, marital status
Świąder et al., [59]	Extrinsic product characteristics	Price
	Intrinsic product characteristics	Nutritional properties, sensory properties
	Sociocultural factors	Consumption habits, cultural influence
Tomic et al., [82]	Intrinsic product characteristics	Nutritional properties, raw material, sensory properties
Torrico et al., [79]	Intrinsic product characteristics	Nutritional properties
Tribst et al., [91]	Intrinsic product characteristics	Processing method, raw material, sensory properties
Tukel and Sengun [73]	Intrinsic product characteristics	Microbial type, sensory properties
Vecchio et al., [18]	Biological & Physiological	Age, gender
	Extrinsic product characteristics	Health claims, price
	Psychological factors	Familiarity
Vitale et al., [53]	Extrinsic product characteristics	Healthy label
	Intrinsic product characteristics	Processing method
Wikandari et al., [71]	Intrinsic product characteristics	Microbial type, sensory properties
Wu et al., [87]	Intrinsic product characteristics	Processing method, raw material, sensory properties

Table S7. Comparison of the proportions of studies that addressed the different factors according to Mojet's model [1].

Factor	Proportion
Biological & Physiological	0.207 b
Extrinsic product characteristics	0.315 b
Intrinsic product characteristics	0.913 a
Psychological factors	0.293 b
Situational factors	0.033 c
Sociocultural factors	0.402 b

Different letters in the same column mean significant differences among the factors according to the chi-square test ($p < 0.05$) of the K proportion test with the Marascuilo procedure.

Table S8. Comparison of the proportions of studies that addressed the Biological and physiological sub-factors according to Mojet´s model [1]

Sub-factor	Proportion
Age	0.789 a
Gender	0.474 b

Different letters in the same column mean significant differences among the sub-factors according to the chi-square test ($p < 0.05$) of the K proportion test with the Marascuilo procedure.

Table S9. Comparison of the proportions of studies that addressed the Extrinsic product characteristics sub-factors according to Mojet´s model [1].

Sub-factor	Proportion
Brand	0.172
Health claims	0.207
Healthy label	0.241
Nutritional facts	0.069
Origin label	0.069
Packaging design	0.103
Price	0.172
Sustainability Claim	0.103
Type of product label	0.345

Table S10. Comparison of the proportions of studies that addressed the Intrinsic product characteristics sub-factors according to Mojet´s model [1].

Sub-factor	Proportion
Health	0.048 d
Microbial type	0.155 d
Nutritional properties	0.214 bcd
Processing method	0.226 bc
Raw material	0.417 b
Sensory properties	0.774 a

Different letters in the same column mean significant differences among the sub-factors according to the chi-square test ($p < 0.05$) of the K proportion test with the Marascuilo procedure.

Table S11. Comparison of the proportions of studies that addressed the Psychological sub-factors according to Mojet´s model [1].

Sub-factor	Proportion
Behavioral control	0.037 b
Emotions and feelings	0.407 a
Familiarity	0.481 a
Neophobia	0.222 ab

Different letters in the same column mean significant differences among the sub-factors according to the chi-square test ($p < 0.05$) of the K proportion test with the Marascuilo procedure.

Table S12. Comparison of the proportions of studies that addressed the Situational sub-factors according to Mojet´s model [1].

Sub-factor	Proportion
Context	0.667
Point of purchase	0.333

Table S13. Comparison of the proportions of studies that addressed the Socio-cultural sub-factors according to Mojet´s model [1].

Sub-factor	Proportion
Attitude	0.486 a
Beliefs	0.216 abc
Concerns	0.054 c
Consumption habits	0.459 ab
Cultural influence	0.243 abc
Educational level	0.135 abc
Income level	0.081 bc
Knowledge	0.081 bc
Living location	0.027 c
Marital status	0.027 c
Occupation	0.054 c

Different letters in the same column mean significant differences among the sub-factors according to the chi-square test ($p < 0.05$) of the K proportion test with the Marascuilo procedure.
